# The Gut Microbiota in Hematologic Malignancies: Mechanisms, Clinical Associations, and Translational Opportunities

**DOI:** 10.3390/nu18091400

**Published:** 2026-04-29

**Authors:** Santino Caserta, Enrica Antonia Martino, Mamdouh Skafi, Ernesto Vigna, Antonella Bruzzese, Nicola Amodio, Marco Fiorillo, Eugenio Lucia, Graziella D’Arrigo, Virginia Olivito, Caterina Labanca, Francesco Mendicino, Maria Eugenia Alvaro, Giovanni Tripepi, Fortunato Morabito, Massimo Gentile

**Affiliations:** 1 Hematology Unit, Department of Onco-Hematology, Azienda Ospedaliera Cosenza, 87100 Cosenza, Italy; enricaantoniamartino@gmail.com (E.A.M.); ernesto.vigna@aocs.it (E.V.); a.bruzzese@aocs.it (A.B.); e.lucia@aocs.it (E.L.); v.olivito@aocs.it (V.O.); c.labanca@aocs.it (C.L.); f.mendicino@aocs.it (F.M.); m.eugenia983@gmail.com (M.E.A.); 2 Emergency and Internal Medicine Department, Saint Joseph Hospital, East Jerusalem 91192, Palestine; mamdouh272844@gmail.com; 3 Department of Experimental and Clinical Medicine, University of Catanzaro, 88100 Catanzaro, Italy; amodio@unicz.it; 4 Department of Pharmacy, Health and Nutritional Science, University of Calabria, 87036 Rende, Italy; marco.fiorillo@unical.it; 5 Istituto di Fisiologia Clinica del CNR di Reggio Calabria, Consiglio Nazionale delle Ricerche, 89124 Reggio Calabria, Italy; graziella.darrigo@cnr.it (G.D.); giovanniluigi.tripepi@cnr.it (G.T.); 6 AIL Sezione di Cosenza, 87100 Cosenza, Italy; f.morabito53@gmail.com

**Keywords:** gut microbiota, hematologic malignancies, dysbiosis, immune–metabolic crosstalk

## Abstract

Hematologic malignancies arise and progress within a systemic ecosystem in which the gut microbiota is an increasingly recognized, partially modifiable component. Across acute leukemias, chronic lymphocytic leukemia, plasma cell disorders, lymphomas, and clonal myeloid neoplasms, human studies consistently report reduced microbial diversity, depletion of barrier-supportive, short-chain fatty acid-producing commensals, and enrichment of Gram-negative, pro-inflammatory, or hospital-adapted taxa. These alterations are associated with pre-leukemic clonal expansion, adverse genetic and immunological features, progression from precursor conditions, and inferior outcomes after chemotherapy, immunochemotherapy, chimeric antigen receptor T-cell therapy, and allogeneic hematopoietic stem cell transplantation. Mechanistic work in animal models and ex vivo systems demonstrates that microbiota-derived signals and metabolites—including Th17/IL-17-skewing consortia and the lipopolysaccharide intermediate ADP heptose sensed by the cytosolic receptor ALPK1—can actively modulate hematopoietic stem and progenitor cell fitness, inflammatory circuits, and malignant cell survival, supporting a causal role in disease biology. At the same time, major knowledge gaps remain because most human cohorts are small, single-center, and cross-sectional, frequently rely on 16S rRNA profiling, and are vulnerable to dietary, geographic, and treatment-related confounding. Within this context, three translational domains appear particularly promising: pharmaco-microbiomics, microbiome-informed risk stratification, and rational microbiota-targeted interventions, particularly diet-based strategies and antimicrobial stewardship. Here, we provide an integrated, disease-spanning synthesis of these data, emphasizing clonal hematopoiesis and myeloid neoplasms as emerging examples of microbiota–marrow crosstalk and outlining practical priorities for embedding microbiome science into future hematologic trials. Routine microbiome profiling or empiric microbiota-directed therapies cannot yet be recommended in everyday hematology practice, but integrating microbiome science into prospective therapeutic and transplant trials offers a realistic path to improved disease modeling, biomarker development, and rational adjunctive strategies to enhance outcomes for patients with hematologic malignancies.

## 1. Introduction

Hematologic malignancies—including leukemias, lymphomas, and multiple myeloma—arise within immune-specialized tissues whose homeostasis depends on dynamic interactions between hematopoietic stem and progenitor cells, stromal niches, and systemic immune regulation. These disorders reflect not only somatic genetic alterations but also host immune responses and environmental exposures. In leukemia, stepwise acquisition of driver mutations during susceptible developmental windows is influenced by immune context and postnatal environmental factors, including infections, which may modulate leukemogenic evolution in predisposed individuals [1,2].

In lymphomas—particularly non-Hodgkin lymphoma and diffuse large B-cell lymphoma (DLBCL)—genetic and epigenetic lesions cooperate with chronic antigenic stimulation, immune dysregulation, and infectious cofactors such as Epstein–Barr virus and Helicobacter pylori [3,4]. Multiple myeloma (MM) evolves from precursor conditions, including monoclonal gammopathy of undetermined significance (MGUS) and smoldering myeloma (SMM), through a series of genetic events that interact with bone marrow microenvironmental signals and immune escape mechanisms [5].

Parallel advances in cancer genomics and tumor immunology have identified the human microbiota as a potentially important modulator of systemic physiology and immune tone. In this context, the human microbiota has emerged as a potentially important modulator of systemic immunity and metabolism. The gastrointestinal microbiota, which harbors the greatest microbial density, consists of complex communities of bacteria, archaea, viruses, and fungi. Although its taxonomic composition varies across individuals, core functional capacities are relatively conserved, supporting functional redundancy within microbial ecosystems [6].

Mechanistic studies, primarily derived from preclinical models, suggest that the microbiota can influence host physiology through immunologic and metabolic pathways. Microbial metabolites—including short-chain fatty acids (SCFAs), bile acid derivatives, indoles, and polyamines—can modulate epithelial integrity, inflammatory signaling, and immune cell differentiation [7]. These pathways have been implicated in the regulation of hematopoietic and immune tone, although direct evidence in humans remains limited. Microbiota-derived signals further influence regulatory T-cell and Th17-cell differentiation, IgA production, and innate immune responses, thereby shaping host–tumor interactions [8]. Through these pathways, the microbiota intersects with bone marrow niche biology, systemic immunity, and tumor–host interactions relevant to leukemogenesis, lymphomagenesis, and myeloma progression.

The microbiota may also contribute to variability in treatment response. Many drugs used in hematologic oncology can be metabolized by gut bacteria, potentially affecting their bioavailability and activity [9]. In addition, microbiota composition has been associated with xenobiotic pathways [10], suggesting that the microbiome represents an additional layer of pharmacologic variability.

Importantly, the relationship between hematologic malignancies and the microbiota is bidirectional. Dysbiosis has been associated with immune dysregulation, infection susceptibility, and adverse outcomes in the context of hematopoietic stem cell transplantation (HSCT). Conversely, microbiota composition is profoundly influenced by chemotherapy, antibiotic exposure, hospitalization, and nutritional factors. These variables represent major confounders and complicate the interpretation of microbiome data in hematologic patients.

Despite growing interest, the current evidence base remains limited by small sample sizes, cross-sectional designs, and substantial methodological heterogeneity, including differences in sequencing approaches and analytical pipelines. As a result, most available human data are associative, and causal relationships remain largely inferred from preclinical models or genetic approaches such as Mendelian randomization.

In this review, we provide an integrated synthesis of current evidence linking the microbiota to hematologic malignancies across the disease continuum—from clonal hematopoiesis and precursor conditions to overt disease and transplantation. We outline key mechanistic pathways of microbiota–immune–metabolic crosstalk, examine disease-specific associations, and discuss emerging translational applications, including pharmacomicrobiomics, risk stratification, and microbiota-targeted interventions, also supported by explanatory figures created with the support of ChatGPT free (GPT-5.3) (OpenAI). Throughout, we emphasize methodological limitations, potential confounders, and priorities for future research needed to translate microbiome science into clinically meaningful strategies in hematology.

## 2. Mechanistic Framework: Microbiota–Immune–Metabolic Crosstalk Relevant to Hematology

The development and steady-state function of the hematopoietic and immune systems are influenced by signals derived from the microbiota, as demonstrated primarily in preclinical models. Germ-free or antibiotic-treated animals exhibit profound defects in immune architecture, including impaired secondary lymphoid organ development, reduced CD4^+^ T-cell and IgA-producing plasma cell numbers, and defective gut-associated lymphoid tissue (GALT) formation; many of these abnormalities are partially restored upon microbial colonization [10,11,12]. Although these findings provide important mechanistic insights, their direct applicability to human hematopoiesis remains incompletely defined.

### 2.1. Microbiota-Dependent Maturation of Immunity and Hematopoiesis

The cytokines, growth factors, and pattern-recognition pathways that regulate mucosal immune maturation also influence bone marrow output and peripheral immune homeostasis. In experimental models, microbiota-derived signals contribute to the regulation of myelopoiesis and neutrophil dynamics. For example, systemic sensing of gut-derived peptidoglycan via NOD1 has been shown to enhance granulopoiesis and neutrophil function, indicating that commensal-derived signals help establish baseline of innate immune activity [13]. Circulating microbial ligands may also regulate monocyte trafficking and steady-state myelopoiesis [14,15]. However, evidence for these mechanisms in humans remains limited.

At the adaptive level, the microbiota appears to play an important role in shaping T-cell ontogeny and subset balance, largely based on animal studies. Segmented filamentous bacteria promote Th17 differentiation, whereas defined consortia, including Clostridium clusters IV and XIVa, induce Foxp3^+^ regulatory T cells [16,17]. Additional microbial products, such as polysaccharide A from *Bacteroides fragilis*, have been shown to promote immune tolerance through Treg-skewing pathways [18], while innate lymphoid cells are similarly influenced by microbial signals [19]. Early-life microbial exposures are considered particularly important in establishing long-term immune tone [20]. Within this framework, Greaves’ “delayed infection” model for childhood acute lymphoblastic leukemia (ALL) proposes that insufficient early immune priming in genetically predisposed children may favor aberrant inflammatory responses later in life [21]. Although biologically plausible, this hypothesis remains largely theoretical in humans, with limited direct clinical evidence.

### 2.2. Microbial Metabolites as Systemic Immunometabolic Regulators

Beyond pattern-recognition signaling, the microbiota exerts systemic effects through small-molecule metabolites that function as host–microbe co-metabolites. These interconnected metabolic pathways link bacterial transformation of dietary and endogenous substrates to bioactive compounds capable of signaling to distal tissues, including bone marrow and lymphoid organs [22]. However, most evidence supporting these systemic effects is derived from experimental models.

SCFAs—including butyrate, propionate, and acetate—are produced by microbial fermentation of complex carbohydrates. These metabolites signal via G protein-coupled receptors and histone deacetylase inhibition, promoting regulatory T-cell differentiation and restraining inflammatory responses [23,24,25]. Preclinical studies suggest that SCFAs may also influence cytokine milieus and hematopoietic stem and progenitor cell (HSPC) behavior, although direct evidence in human bone marrow remains limited.

Additional microbiota-derived pathways include bile acid metabolism, tryptophan-derived indoles, and vitamin synthesis. Secondary bile acids act through receptors such as FXR and TGR5 to modulate metabolic and immune functions, while indoles engage the aryl hydrocarbon receptor, influencing barrier integrity and immune homeostasis [7,22].

Microbial production of vitamins, including folate and other B vitamins, further supports DNA synthesis, epigenetic regulation, and hematopoiesis [26].

Alterations in these pathways during dysbiosis may promote pro-inflammatory states relevant to hematologic malignancies. Consistently, disruptions in SCFAs, bile acids, and tryptophan metabolism have been reported in hematologic diseases and are associated with disease progression, although causality remains to be established.

Overall, microbiota-derived metabolites represent a biologically plausible interface between microbial ecosystems and hematopoietic regulation. Nevertheless, their precise role in human hematologic malignancies remains incompletely defined and requires validation in well-designed longitudinal and interventional studies.

### 2.3. Intestinal Barrier Integrity, Dysbiosis, and Inflammatory Signaling

The intestinal mucosal barrier constitutes a multilayered defense system that separates luminal microbes from host tissues [10]. Secretory IgA and antimicrobial peptides such as RegIIIγ limit bacterial adhesion and translocation [27,28], while tonic Toll-like receptor signaling supports epithelial repair and homeostasis [29]. Disruption of this barrier—through infection, diet, chemotherapy, irradiation, or antibiotic exposure—can promote dysbiosis and microbial translocation. Experimental models show that barrier disruption and inflammasome dysfunction can promote intestinal permeability and systemic inflammation, including IL-6- and TNF–α-driven pathways [30,31]. In humans, chronic low-grade endotoxemia has been associated with inflammatory states [32]. Inflammatory cytokines such as IL-6, TNF-α, and IL-1β are established contributors to hematologic malignancy biology [33]. These observations suggest that microbiota-mediated barrier dysfunction may contribute to pro-inflammatory environments relevant to hematologic malignancies, although direct causal evidence in patients remains limited.

### 2.4. Therapy- and Antibiotic-Induced Microbiota Perturbation in Hematologic Oncology

Cytotoxic therapy, conditioning regimens, HSCT, immunotherapies, and broad-spectrum antibiotics profoundly alter the gut microbiota in hematologic patients. These interventions typically reduce microbial diversity and promote domination by single taxa, often *Enterococcus*, or members of the Enterobacteriaceae family. In observational studies, such changes have been associated with bacteremia, GVHD, and mortality following allo-HSCT [34,35]. These treatment-related perturbations represent major confounding factors when interpreting microbiome–disease associations. Such treatment-induced ecological shifts reflect and reinforce the mechanisms outlined above: depletion of SCFA-producing commensals, disruption of barrier function, and expansion of pathobionts capable of translocation and systemic inflammation. The resulting IL-6- and TNF–α-rich environment can influence leukemic cell survival and chemoresistance, while overgrowth of antibiotic-resistant taxa increases infectious morbidity and constrains antimicrobial choices.

Overall, microbiota-dependent regulation of hematopoiesis, systemic metabolite signaling, and barrier integrity provides a conceptual framework through which microbial ecosystems may influence the initiation, progression, and therapeutic response of hematologic cancers. However, much of this framework is derived from preclinical models, and its translation to human disease remains incomplete. Future studies integrating mechanistic, longitudinal, and interventional approaches will be essential to clarify the clinical relevance of these pathways.

**Figure 1:**
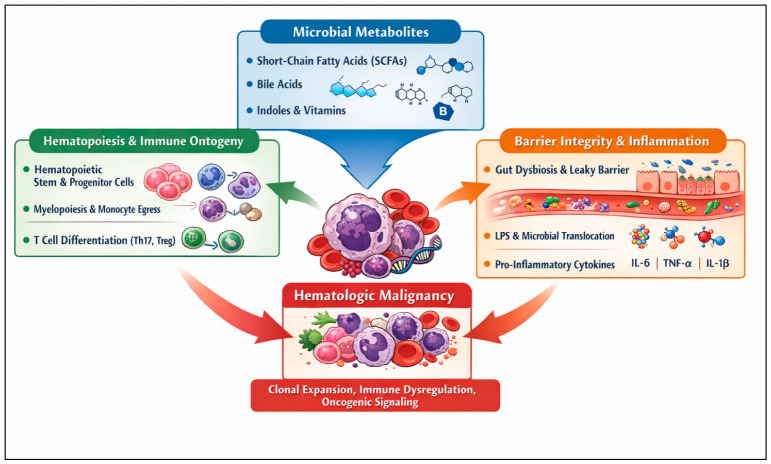
Principal microbiota–immune–metabolic crosstalk in hematologic malignancies. Microbial metabolites produced by the gut microbiota, including short-chain fatty acids (SCFAs), bile acids, indoles, and vitamins (top, blue box), act on host tissues and immune cells to influence hematologic cancer. On the left (green box), these metabolites regulate hematopoiesis and immune ontogeny by modulating hematopoietic stem and progenitor cells, myelopoiesis and monocyte egress, T-cell differentiation toward Th17, and regulatory T (Treg) subsets. On the right (orange box), dysbiosis and impaired barrier integrity promote luminal lipopolysaccharide (LPS) and microbial translocation, driving the production of pro-inflammatory cytokines such as IL-6, TNF-α, and IL-1β. Together, these alterations converge on hematologic malignancy (central and bottom red panels), fostering clonal expansion of malignant cells, immune dysregulation, and oncogenic signaling.

These principal microbiota-mediated mechanisms are schematically summarized in Figure 1 and provide the biological context for the disease-specific associations discussed in subsequent sections. Across entities, a recurring pattern emerges in which loss of SCFA-producing commensals, Gram-negative overgrowth, and barrier dysfunction converge on chronic IL-6/IL-1β/TNF-α signaling and altered HSPC fitness, offering multiple potential points for therapeutic intervention.

## 3. Disease-Specific Microbiota Alterations in Hematologic Malignancies

Hematologic malignancies encompass a heterogeneous group of disorders in which genetic predisposition, environmental exposures, and immune regulation converge to shape disease onset, progression, and therapeutic response [36]. Across entities, most human studies—despite being small, heterogeneous, and often cross-sectional—converge on a recurring dysbiotic pattern: reduced α diversity, depletion of barrier-supportive, SCFA-producing commensals (e.g., many *Lachnospiraceae* and *Ruminococcaceae*), and enrichment of Gram-negative, pro-inflammatory, or hospital-adapted taxa. These shifts are variably linked to pre-leukemic clonal expansion, adverse genetic and immunologic features, progression from precursor states, infectious complications, and inferior outcomes after chemotherapy, immunotherapy, CAR T-cell therapy, and HSCT.

### 3.1. Acute Leukemias

The microbiota has been increasingly implicated in acute leukemias, particularly acute myeloid leukemia (AML) and acute lymphoblastic leukemia (ALL). However, in humans, most available data are associative and derived from small, heterogeneous cohorts. Reported microbiome alterations must also be interpreted in light of major confounding factors, including antibiotic exposure, chemotherapy, hospitalization, and nutritional status.

#### 3.1.1. Composition and Diversity

In childhood precursor B-ALL, multistep leukemogenesis involves prenatal genetic lesions (e.g., ETV6–RUNX1, hyperdiploidy) that generate clinically silent pre-leukemic clones requiring postnatal “second hits” [37]. Early-life microbial exposures and infection patterns have been proposed as critical environmental modulators within this “delayed infection” or “two-step” model. Consistent with this framework, several 16S rRNA and metagenomic studies report reduced α-diversity and altered community structure in children with newly diagnosed ALL compared with healthy controls, characterized by depletion of SCFA-producing taxa and enrichment of opportunistic organisms; induction chemotherapy and antibiotics further accentuate these changes [38].

In AML, several studies have reported reduced microbial diversity and compositional shifts, often characterized by decreased abundance of commensal anaerobes and expansion of opportunistic taxa, including *Enterococcus* and members of the Enterobacteriaceae family [39]. These patterns have been associated with infectious complications and adverse outcomes. However, most studies are cross-sectional, include limited sample sizes, and are heavily influenced by treatment-related exposures, making it difficult to disentangle disease-specific signals from therapy-induced dysbiosis.

#### 3.1.2. Mechanistic and Causal Support

Experimental models support a causal role for the microbiota in leukemogenesis. In Pax5^+/−^ mice, early-life broad-spectrum antibiotics disrupt the gut microbiome and significantly increase precursor B-ALL incidence, even without exogenous infectious challenges; distinct microbial signatures distinguish animals that develop leukemia from those that remain disease-free [40]. These data are consistent with the idea that microbiota-shaped immune ontogeny (e.g., Th17/Treg balance, cytokine milieu, barrier integrity) modulates the likelihood that pre-leukemic clones progress to overt ALL.

Importantly, most causal evidence supporting a microbiota–leukemia link all derives from preclinical models. While these studies provide important mechanistic insights, they cannot be directly extrapolated to human disease. In humans, the available evidence remains largely observational and cross-sectional, and therefore primarily supports associations rather than causality.

Mendelian randomization (MR) analyses add a genetic layer of support. Using large GWAS datasets, Chen et al. identified specific microbial clades with putative causal associations with leukemia risk, with some *Firmicutes*/*Clostridia* lineages appearing protective and *Gammaproteobacteria*-associated taxa conferring increased risk [41]. Mendelian randomization analyses provide a genetic framework to explore potential causal relationships; however, they remain indirect inferential approaches and cannot substitute for prospective human studies or clinical trials.

#### 3.1.3. Clinical Correlations and Transplant Setting

In patients receiving intensive therapy for AML and in allo-HSCT recipients, treatment-induced dysbiosis has clear clinical consequences. Intestinal domination by *Enterococcus* or Enterobacteriaceae and severe loss of diversity during induction chemotherapy or conditioning are consistently linked to increased bloodstream infections and adverse outcomes [23,34]. In a four-center cohort of 1362 allograft recipients, low fecal diversity and domination by single taxa (commonly *Enterococcus*, *Streptococcus*, or *Proteobacteria*) around neutrophil engraftment independently predicted higher non-relapse and overall mortality, particularly GVHD- and infection-related deaths [42]. More recently, early post-transplant stool features—severe diversity loss, frequent *Enterococcus*/Enterobacteriaceae domination, and enrichment of *Collinsella* and *Eggerthella*—predicted acute and chronic GVHD, relapse, and survival in adult and pediatric cohorts [43]. By contrast, a Brazilian study reported different domination fingerprints without clear outcome associations, underscoring geographic and dietary context [44].

Interpretation of microbiome alterations in acute leukemia requires caution, as many observed changes may be influenced by intensive chemotherapy, hospitalization, broad-spectrum antibiotic exposure, and nutritional alterations during treatment. These factors may significantly shape gut microbial composition and complicate attribution of dysbiosis directly to leukemia biology.

#### 3.1.4. Summary and Implications

**Figure 2:**
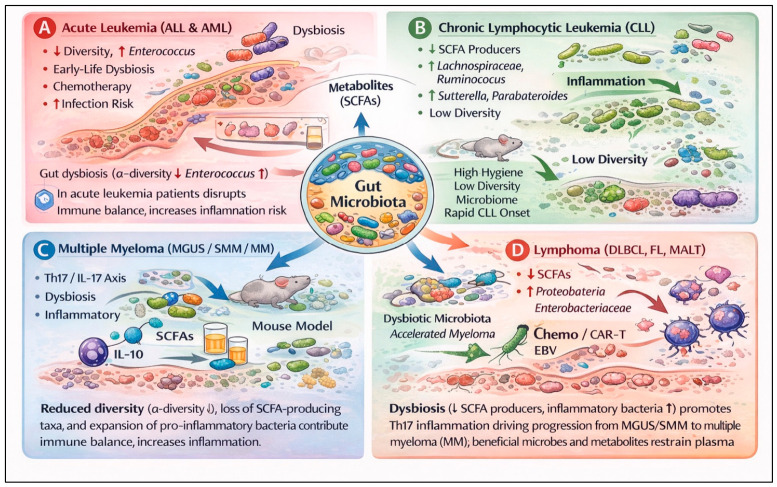
Disease-specific microbiota–immune mechanisms in hematologic malignancies. Schematic overview of how alterations in gut microbiota influence immune responses across different hematologic malignancies. Panel (**A**)—Acute Leukemia (ALL and AML): Early-life dysbiosis, chemotherapy, and infections disrupt microbial diversity and increase *Enterococcus* abundance, leading to impaired immune balance and higher infection risk. Reduced α-diversity is associated with elevated inflammation and compromised gut barrier function [41]. Panel (**B**)—Chronic Lymphocytic Leukemia (CLL): Low microbial diversity, loss of SCFA-producing taxa, and enrichment of *Lachnospiraceae* and *Ruminococcus* drive inflammatory responses. These microbiota changes correlate with disease progression and immune dysregulation [45]. Panel (**C**)—Multiple Myeloma (MGUS/SMM/MM): Dysbiosis and loss of SCFA-producing bacteria shift the Th17/IL-17 axis toward pro-inflammatory signaling. Mouse models demonstrate that reduced SCFA availability increases inflammation, while IL-10 and metabolites help restrain plasma cell proliferation [21]. Panel (**D**)—Lymphoma (DLBCL, FL, MALT): Expansion of *Proteobacteria* and Enterobacteriaceae, alongside reduced SCFA producers, contributes to inflammatory signaling. Chemotherapy, CAR-T therapy, and EBV infection further exacerbate dysbiosis, influencing disease course and treatment outcomes [46]. Central Mechanism: Across malignancies, gut microbiota dysbiosis alters metabolite production (notably SCFAs), disrupts immune homeostasis, and modulates inflammation, collectively affecting disease progression and therapy response.

In acute leukemias, the gut microbiota appears to operate at three levels: shaping early-life immune ontogeny and pre-leukemic clone evolution; modulating chemotherapy-related toxicity, infection risk, and dysbiosis during induction; and influencing allo-HSCT outcomes via diversity loss and domination patterns. Diversity and domination indices around engraftment consistently emerge as robust prognostic markers, although the specific high-risk taxa vary with geography, diet, and antibiotic exposure (Figure 2A, Table 1). Despite the growing number of studies describing microbiota alterations in acute leukemias, most available human data derive from relatively small cohorts and are frequently cross-sectional, limiting causal inference. Many analyses rely on 16S rRNA profiling and may be influenced by confounding factors such as antibiotic exposure, diet, hospitalization, and chemotherapy. Consequently, although associations between dysbiosis, infection risk, and treatment outcomes are increasingly reported, the clinical relevance and mechanistic directionality of these findings remain to be confirmed in larger prospective studies. Taken together, these data support the concept that acute leukemia is not only a genetic disease of hematopoietic precursors but also a disorder whose clinical course is modulated by the surrounding intestinal ecosystem.

### 3.2. Chronic Lymphocytic Leukemia

#### 3.2.1. Composition and Diversity

Cross-sectional fecal microbiome profiling consistently shows that patients with CLL harbor reduced α diversity and altered community structure compared with healthy controls. Studies report depletion of SCFA-producing taxa (e.g., *Lachnospiraceae*, *Ruminococcaceae*) and enrichment of taxa linked to dysbiosis, including *Bacteroides*, *Parabacteroides*, and *Sutterella*, alongside underrepresentation of genera such as *Bifidobacterium* and *Anaerostipes* [45]. At higher taxonomic levels, increased *Firmicutes*/*Bacteroidota* ratios and *Proteobacteria* enrichment correlate with adverse prognostic features—unmutated IGHV, high CD38 expression—and shorter time to first treatment, suggesting that phylum-level shifts track with disease severity and progression [47].

#### 3.2.2. Mechanistic and Causal Support

Deeper metagenomic profiling in a larger cohort (*n* = 59) showed that microbiome diversity clusters map onto heterogeneous clinical courses: patients with the lowest diversity have more advanced disease, more prior treatment, and more progression events than those with higher diversity [45]. Shotgun data identified taxonomic and functional signatures associated with more aggressive CLL.

Mechanistic evidence comes from the Eµ-TCL1 mouse model. Mice housed under high-hygiene conditions develop CLL more rapidly and harbor gut microbiota dominated by genera such as *Mucispirillum* and *Parabacteroides*, whereas mice with higher microbiome diversity experience slower disease kinetics; microbiome diversity thus appears to modulate leukemia development, supporting a causal role rather than a mere association [45]. Although these findings suggest that microbiome composition may influence leukemia kinetics in experimental systems, it should be emphasized that such causal relationships have not yet been demonstrated in human CLL. Current human studies remain observational and are therefore insufficient to establish a direct causal role of dysbiosis in disease progression.

#### 3.2.3. Clinical Correlations and Implications

Clinically, microbiome features in CLL correlate with disease stage, biological risk markers, and time to first treatment. Patients with CLL show reduced diversity and depletion of putative SCFA-producing commensals compared with controls, with more pronounced alterations in advanced disease and in previously treated patients [45]. Phylum-level changes (increased *Firmicutes*/*Bacteroidota* ratios, *Proteobacteria* enrichment) are associated with unmutated IGHV, high CD38, and shorter time to first treatment [47]. Together, the consistent association of low diversity and relative loss of SCFA-producing taxa with more advanced or high-risk CLL supports the notion that dysbiosis may exacerbate the chronic inflammatory and immunoregulatory disturbances inherent to this disease [45]. Data directly linking specific microbiome configurations to responses or toxicities under defined therapeutic regimens (e.g., BTK or BCL2 inhibitors) remain sparse and warrant dedicated prospective studies.

In addition, several non-disease-related factors—including age, comorbidities, diet, medication exposure (including proton pump inhibitors and antibiotics), and prior treatments—may influence microbiome composition in *CLL* patients and should be considered when interpreting reported associations.

#### 3.2.4. Summary and Implications

In CLL, cross-sectional and experimental data indicate that low microbial diversity and depletion of SCFA-producing taxa are linked to more advanced disease, high-risk biological markers, and faster need for treatment. Although causality in humans remains to be proven, the Eµ TCL1 model suggests that microbiome configuration can influence leukemia kinetics. Current evidence linking the gut microbiome to CLL biology is still limited and largely based on small observational cohorts with heterogeneous designs. Most studies are cross-sectional and include patients with different disease stages and treatment histories, introducing potential confounding that complicates interpretation. While consistent patterns of reduced diversity and altered taxonomic composition have been described, the strength of evidence supporting a causal role of dysbiosis in CLL progression remains modest and requires validation in larger, longitudinal studies. Figure 2B and Table 2 summarize gut microbiota alterations in CLL, highlighting mechanistic insights and clinical correlations.

### 3.3. Multiple Myeloma and Monoclonal Gammopathies (MGUS/SMM)

#### 3.3.1. Composition and Diversity

Observational 16S rRNA and metagenomic studies show that MM patients harbor a distinct gut microbiome compared with healthy controls, with reduced diversity, depletion of SCFA-producing commensals (e.g., many *Ruminococcaceae*, *Lachnospiraceae*), and enrichment of pro-inflammatory/opportunistic taxa, including *Proteobacteria*, *Streptococcus*, *Klebsiella*, and *Pseudomonas* [2,21]. In a Chinese cohort, increased *Pseudomonas aeruginosa* and altered *Faecalibacterium* were observed; higher *Faecalibacterium prausnitzii* levels correlated with ISS stage, underscoring context-dependent roles even within SCFA producers [2]. Similar dysbiotic features appear detectable at the MGUS/SMM stage and may relate to progression risk [2].

#### 3.3.2. Mechanistic and Causal Support

Preclinical work has provided proof of principle that specific microbes can accelerate myeloma evolution. In Vk*MYC models, colonization with *Prevotella heparinolytica* drives gut Th17 responses and homing of IL-17-producing cells to the bone marrow, where they activate eosinophils and IL-6 production, thereby accelerating progression from an MGUS/SMM-like phase to overt MM; IL-17 blockade or microbiota disruption delays disease onset [48]. These experimental findings provide biologic plausibility for a microbiota–myeloma axis; however, they originate primarily from animal models and controlled experimental settings. In human cohorts, evidence linking specific microbial taxa to disease progression remains largely associative and should therefore be interpreted cautiously. In patients with SMM, higher bone marrow IL-17 levels similarly predicted faster progression in the same study.

Conversely, some microbiota-derived metabolites have anti-myeloma properties. Rodríguez-García et al. showed that MGUS or treated MM patients with urolithin-producing taxa and detectable urolithin A have better outcomes; urolithin A is cytotoxic to MM cell lines, inhibits primary MM cell proliferation ex vivo, and synergizes with bortezomib in xenograft models, including bortezomib-resistant disease [49]. A two-sample MR analysis further suggested that higher genetically predicted abundance of some genera (e.g., *Eubacterium ruminantium* group) may increase MM risk, whereas *Dorea*, *Coprococcus*, and several *Ruminococcaceae* taxa may be protective, although these signals require replication.

#### 3.3.3. Clinical Correlations, Diet, and Transplantation

Interventional data support partial modifiability of this axis. In the NUTRIVENTION trial (NCT04920084), a whole-food, high-fiber, plant-based diet plus behavioral coaching in individuals with MGUS/SMM and elevated BMI improved modifiable risk factors (BMI, insulin resistance, systemic inflammation, monocyte subsets) and increased microbiome diversity and SCFA production; disease trajectories were stabilized or improved over 36 weeks. Around autologous and allo-HSCT in MM, small studies document marked loss of diversity during conditioning and engraftment, transient overgrowth of *Bacteroides*, *Enterococcus*, and other hospital-associated taxa, and associations between microbiome features and neutrophil engraftment, mucosal toxicity, neutropenic fever, and bacteremia [2].

Interpretation of microbiome findings in plasma cell disorders should also account for potential confounders such as diet, metabolic status, antibiotic exposure, and treatment-related factors. These variables may independently influence microbial composition and may partially explain differences observed between patient groups.

#### 3.3.4. Summary and Implications

Data on MGUS, SMM, and MM converge on a coherent picture (Figure 2C, Table 3): dysbiosis—characterized by loss of SCFA-producing commensals and expansion of pro-inflammatory taxa—contributes to a Th17-skewed, metabolically perturbed microenvironment that promotes plasma cell growth and accelerates progression from precursor states. At the same time, specific bacteria and metabolites (e.g., urolithin-producing taxa, butyrate producers enriched by high-fiber diets) appear capable of restraining this process. Evidence connecting the microbiota to the myeloma disease continuum is supported by both observational human studies and mechanistic data from preclinical models. However, many clinical studies remain small and methodologically heterogeneous, with differences in sequencing approaches, patient populations, and control selection. As a result, although links between dysbiosis, Th17-skewed inflammation, and disease progression are biologically plausible, the translational and clinical significance of these associations should be interpreted cautiously pending confirmation in larger prospective cohorts and interventional trials. The NUTRIVENTION study provides early proof of principle that structured nutritional interventions can reproducibly shift the microbiome, improve systemic inflammatory and metabolic profiles, and potentially stabilize disease trajectories.

### 3.4. Lymphoma

#### 3.4.1. Composition and Diversity

Long-standing paradigms illustrate microbe-driven lymphomagenesis, notably *H. pylori* in gastric MALT lymphoma and EBV in several Hodgkin and non-Hodgkin lymphomas [46,53,54]. More recently, attention has turned to the broader gut microbiota, particularly in DLBCL and follicular lymphoma (FL).

In untreated DLBCL, multiple 16s rRNA studies report reduced α-diversity and distinct taxonomic signatures compared with controls, with enrichment of *Proteobacteria* and Enterobacteriaceae and depletion of SCFA-producing genera such as *Faecalibacterium*, *Roseburia*, and *Lachnospiraceae* [55,56]. A 2025 study comparing primary and relapsed/refractory (RR) DLBCL and controls found fusobacteria enrichment in primary disease, with rr cases showing decreased fusobacterium and increased enterococcus; cytochrome p450-related functions were upregulated in RR-DLBCL [57].

In FL, Xu et al. reported a distinct fecal profile vs. controls, with higher α diversity, enrichment of *Ruminococcaceae* and reduction in *Coriobacteriaceae* [57]. Duodenal mucosal samples from gastrointestinal FL show reduced α diversity and altered community structure [58]. Across B-cell non-Hodgkin lymphomas, a continuum of dysbiosis is seen, with DLBCL exhibiting more pronounced alterations than FL or marginal zone lymphoma [59].

#### 3.4.2. Mechanistic and Causal Support

Elkourashy et al. integrated observational and mr data to examine gut dysbiosis in DLBCL [60]. Pro-inflammatory and bile acid-modifying taxa (e.g., *Bilophila*, *Desulfovibrionaceae*, *Coprobacter*) were enriched in DLBCL, whereas taxa with anti-inflammatory or metabolic-regulatory roles (*Eubacterium coprostanoligenes* group, *Alistipes*, *Ruminococcaceae* Ucg-011) were depleted. Mr signals, although modest, are biologically coherent and support roles for specific commensals in lymphomagenesis via lipid metabolism, bile acid transformation, barrier integrity, and systemic inflammation.

Microbiota-derived SCFAs and other metabolites modulate T-cell differentiation, germinal-center dynamics, and systemic inflammation, providing a plausible link between gut ecology and FL/DLBCL-relevant immune circuits [61,62]. MR data also suggest that some *Ruminococcaceae* taxa may be causally related to FL risk, although results are not yet consistent across subtypes [56].

#### 3.4.3. Clinical Correlations, Immunochemotherapy, and CAR T

In treatment-naïve DLBCL, dysbiosis has been associated with aggressive histology and adverse clinical outcome [59]. During R-CHOP-based immunochemotherapy, baseline microbiome composition correlates with efficacy and toxicity: enrichment of proteobacteria and inflammatory taxa associates with inferior response and increased infectious complications, while treatment-induced enrichment of Enterobacteriaceae parallels infection risk [63].

In RR-DLBCL treated with CD19-directed CAR T-cell therapy, baseline abundance of *Bacteroides fragilis* is enriched in responders and correlates with longer progression-free survival; functional profiling reveals enrichment of inosine biosynthesis pathways, and circulating inosine correlates with both B. fragilis and outcome. Patients with higher baseline abundance of SCFA producers (*Bifidobacterium*, *Faecalibacterium*, *Roseburia*) have lower rates of cytokine-release syndrome and neurotoxicity, whereas branched-chain fatty acid pathways are enriched in those developing toxicity [63].

In FL, higher relative abundance of *Ruminococcus* (within *Ruminococcaceae*) is associated with higher tumor burden, elevated LDH, and trends toward higher FLIPI and grade, suggesting that specific *Ruminococcaceae* clades may mark a more aggressive phenotype, though sample size and 16S rRNA resolution limit conclusions [38].

In lymphoma cohorts, microbiome composition may also be affected by treatment-related variables—including chemotherapy, immunotherapy, antibiotic exposure, and hospitalization—making it difficult to disentangle disease-associated dysbiosis from treatment-induced microbial shifts.

#### 3.4.4. Summary and Implications

Across lymphomas, particularly DLBCL and to a lesser extent FL, reproducible dysbiotic signatures—loss of SCFA-producing commensals and expansion of inflammatory or bile acid-modifying taxa—correlate with histologic aggressiveness, outcomes, and responses/toxicities to immunochemotherapy and CAR T-cell therapy (Figure 2D, Table 4). Studies evaluating the microbiome in lymphoma are comparatively limited and often involve heterogeneous disease entities, treatment settings, and sampling strategies. Most available data are derived from small cross-sectional analyses, which restrict the ability to distinguish whether dysbiosis represents a driver of disease biology or a consequence of treatment, systemic inflammation, or environmental exposures. Therefore, although emerging data suggest potential interactions between microbial composition, immune signaling, and lymphoma outcomes, the current level of evidence remains preliminary. Notably, the association of *Bacteroides fragilis* and inosine pathways with CAR T-cell efficacy, and of SCFA producers with reduced toxicity, mirrors findings from solid-tumor immunotherapy and suggests convergent microbiome–immunotherapy interactions across oncology.

### 3.5. Other Hematologic Entities: MDS, MPNs, and CHIP

#### 3.5.1. Composition and Diversity

MDS and Philadelphia-negative MPNs (polycythemia vera, essential thrombocythemia, primary myelofibrosis) are clonal hematopoietic disorders driven by somatic mutations in stem and progenitor cells, but differ in ineffective vs. proliferative hematopoiesis and clinical features [56,64].

In both, the gut microbiota is increasingly recognized as a relevant extrinsic determinant of clonal behavior, inflammatory tone, and progression risk.

In MDS, Riello et al. profiled fecal 16S rRNA sequences from 30 patients and 16 elderly controls and found dysbiosis that tracked with risk category: high-risk and excess-blast MDS were enriched in Gram-negative *Prevotella* spp. and depleted in *Akkermansia* and *Ruminococcus*, consistent with a shift toward pro-inflammatory, LPS-rich communities in more advanced disease [65]. Jiang et al. combined microbiome and plasma metabolomics and reported enrichment of *Haemophilus parainfluenzae*, *Streptococcus*, and *Clostridium citroniae* and depletion of *Prevotella copri*, alongside distinct metabolomic alterations and correlations with lymphocyte subsets [66].

In MPNs, several cohorts now report distinct microbiota signatures. In PV, reduced α diversity and lower *Firmicutes* abundance—particularly butyrate producers such as *Faecalibacterium*, *Ruminococcaceae*, and *Lachnospiraceae*—have been observed; microbiota composition varies by treatment and appears more “normalized” under interferon-α2 than hydroxyurea or no therapy [67]. In a mixed MPN cohort, disease status explained a small but significant fraction of variance, with fewer reads mapping to an anti-inflammatory *Phascolarctobacterium* and positive correlations between *Parabacteroides* and plasma TNF-α. An ET cohort showed pronounced alterations, especially in JAK2V617F-positive disease, again centered on loss of SCFA-producing *Firmicutes* [67].

At the precursor level, CHIP is increasingly linked to age-related alterations in the gut–bone marrow axis [68]. Agarwal et al. showed that intestinal barrier disruption and dysbiosis—particularly expansion of Gram-negative bacteria—promote selective expansion of Dnmt3a- and Tet2-mutant HSCs in mice, and that fecal microbiota transfer from inflamed or aged donors drives similar effects [69]. Human plasma from individuals with CHIP, MDS, and inflammatory bowel disease shows elevated levels of the LPS biosynthetic intermediate ADP heptose, a key microbial metabolite in this axis.

#### 3.5.2. Mechanistic and Causal Support

Agarwal et al. demonstrated that systemic ADP heptose is sensed by the cytosolic receptor ALPK1, which signals via TIFA and UBE2N to activate NF-κB in HSCs. Dnmt3a-mutant HSCs exhibit ALPK1 promoter hypomethylation and increased expression, rendering them hypersensitive to ADP heptose; genetic ablation of Alpk1 or pharmacologic inhibition of UBE2N prevented clonal expansion [69]. These findings establish a direct mechanistic link between aging-associated gut leakiness, microbiota-derived metabolites, and the competitive advantage of mutant HSC clones.

In MDS, a bidirectional two-sample MR study suggested that genetically predicted abundance of several taxa (e.g., *Blautia*, *Intestinibacter*, *Ruminococcaceae* UCG-003) may increase MDS risk, whereas the classes *Clostridia*, *Veillonellaceae*, *Coprococcus*, and *Clostridiales* are protective; part of the microbiota effect is mediated via specific immune cell phenotype [51]. In MPNs, MR and mediation analysis suggest causal effects of certain gut taxa and plasma metabolites on MPN risk, with part of the microbiota effect transmitted via pro-inflammatory and oxidative stress-related metabolites [52].

Dietary intervention data, though limited, support partial reversibility. In a Mediterranean diet intervention for 28 MPN patients, modest overall diversity changes but shifts in individual taxa and inferred functions were consistent with reduced inflammation [70]. Fecal microbiota transplantation from young into aged mice rejuvenates aged HSCs and suppresses inflammation [71], suggesting that microbiota-directed strategies may influence age-related clonal dynamics.

Moreover, patients with clonal myeloid disorders are often older and frequently exposed to multiple medications and comorbid conditions, which may independently influence microbiome composition. These potential confounders should be considered when interpreting reported microbiota alterations.

#### 3.5.3. Summary and Implications

**Figure 3:**
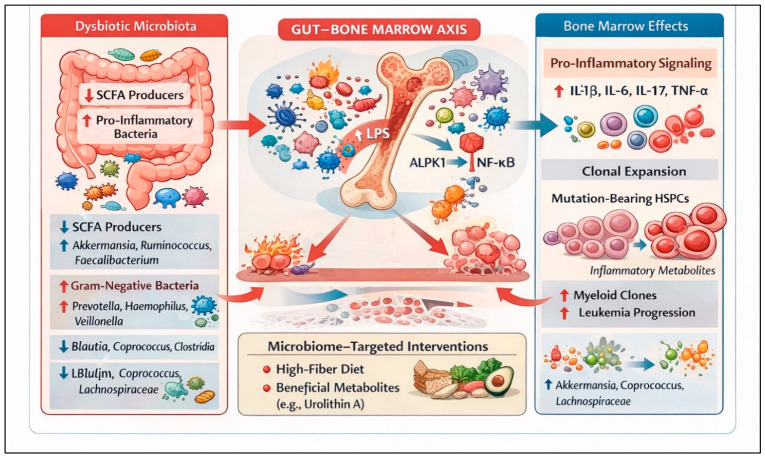
Gut–bone marrow axis in clonal hematopoiesis, myelodysplastic syndromes, and myeloproliferative neoplasms. The schematic illustrates how dysbiotic gut microbiota may influence clonal hematopoiesis and disease evolution in MDS/MPN. **Left**: Intestinal dysbiosis is characterized by loss of short-chain fatty acid (SCFA)-producing commensals and expansion of pro-inflammatory, often Gram-negative, bacteria, with shifts in specific genera (e.g., reduced beneficial taxa and increased pathobionts). **Middle**: Barrier dysfunction and microbial imbalance promote translocation of bacterial products such as lipopolysaccharide (LPS) and other inflammatory metabolites into the circulation, where they signal to the bone marrow. Engagement of pattern-recognition pathways, including ALPK1–NF-κB signaling, skews the marrow microenvironment toward a pro-inflammatory state. **Right**: This inflammatory milieu (elevated IL-1β, IL-6, IL-17, TNF-α) enhances the fitness and clonal expansion of mutation-bearing hematopoietic stem and progenitor cells (HSPCs), fostering myeloid clone outgrowth and progression toward overt leukemia. Bottom: Microbiome-targeted interventions—such as high-fiber, plant-forward diets and supplementation with beneficial microbial metabolites (e.g., urolithin A)—may enrich protective taxa (e.g., *Akkermansia*, *Coprococcus*, *Lachnospiraceae*), restore SCFA production, and potentially mitigate inflammatory signaling along the gut–bone marrow axis.

In CHIP, MDS, and MPNs, the microbiome appears to influence clonal hematopoiesis and disease evolution via Gram-negative overgrowth, barrier dysfunction, and chronic inflammatory signaling. Mechanistic work around ADP heptose–ALPK1–UBE2N–NF-κB signaling provides a concrete example of how a specific microbial metabolite can preferentially expand mutant HSCs. Human MDS and MPN cohorts consistently show reduced diversity, loss of SCFA-producing *Firmicutes*, and enrichment of pro-inflammatory taxa correlated with risk categories and cytokine profiles. MR studies and early dietary interventions further support a causal and modifiable gut–marrow axis. While it is premature to implement microbiome-based interventions for CHIP/MDS/MPN in routine care, these entities offer an attractive setting for preventive and disease-modifying strategies targeting intestinal barrier integrity, Gram-negative dysbiosis, and inflammaging (Figure 3, Table 5). Available studies are few, typically involve small cohorts, and often rely on cross-sectional designs with limited control for confounding factors, including age, comorbidities, and medication use. Consequently, while preliminary observations suggest potential links between dysbiosis, systemic inflammation, and clonal hematopoiesis, the strength of evidence remains limited and should be interpreted as hypothesis-generating rather than definitive. Because clonal hematopoiesis is common in the aging population, even modest microbiota-mediated effects on clone expansion could have a substantial population-level impact, underscoring the need for carefully designed interventional studies in this space.

## 4. Translational Applications and Future Directions

The growing body of evidence linking the gut microbiota to hematologic malignancies has generated increasing interest in its potential clinical applications, including microbiome-informed risk stratification, prediction of treatment response and toxicity, and the development of microbiota-targeted interventions. However, despite strong biological plausibility and supportive preclinical data, translation into routine clinical practice remains limited.

A first domain is pharmacomicrobiomics. Microbiota–drug interactions add a layer to host pharmacogenetics: gut bacteria can directly transform many orally administered agents or modulate hepatic xenobiotic pathways, thereby influencing systemic exposure, efficacy, and toxicity [7,9]. Across hematologic entities, altered xenobiotic metabolism signatures in relapsed/refractory DLBCL, treatment-specific microbiome patterns in PV and other MPNs, and profound shifts in microbial metabolic capacity around HSCT are consistent with a pharmacomicrobiomic contribution to interpatient variability [38,63,67]. Embedding systematic microbiome and metabolome profiling, alongside pharmacokinetics and toxicity phenotyping, into trials of key regimens (anti-CD20-containing combinations, venetoclax- and hypomethylating-based therapies, JAK inhibitors, CAR T-cell therapy, allo-HSCT conditioning, and GVHD prophylaxis) should help identify microbial genes, pathways, or configurations that predict altered drug handling and toxicity, and guide dose optimization and supportive-care adjustments. Such studies should predefine microbiome endpoints, include serial sampling, and integrate host pharmacogenetic data to disentangle microbial from germline contributions to variability.

A second domain is the use of microbiome features for risk stratification and biomarker development. Simple metrics such as α diversity and intestinal domination already show prognostic value in allo-HSCT, where severe diversity loss and domination by single taxa around engraftment predict bacteremia, GVHD, non-relapse mortality, and overall survival [34,42], and longitudinal modeling of early post-transplant trajectories further improves prediction [72]. In precursor states and chronic malignancies, microbiome signatures correlate with progression and high-risk biology, including Th17-skewing communities and IL-17 in MGUS/SMM [48,51], and reduced diversity with adverse markers in CLL, FL, and DLBCL [45,67]. In relapsed/refractory DLBCL treated with CD19-directed CAR T-cell therapy, baseline enrichment of *Bacteroides fragilis* and inosine-producing pathways correlates with longer progression-free survival, while higher abundance of SCFA-producing taxa associates with lower rates of cytokine-release syndrome and neurotoxicity [63], echoing observations in solid tumors [62,73]. In the medium term, such microbiome metrics are most likely to be useful as components of composite prognostic models—integrated with clinical, genomic, immune, and metabolomic parameters—rather than standalone tests. Standardization of sampling, sequencing, and bioinformatic pipelines across centers will be essential if such biomarkers are to transition from exploratory research tools to clinically actionable assays.

A third domain involves microbiota-targeted interventions. Among these, dietary modulation currently has the strongest balance of biologic plausibility, safety, and early clinical support. In NUTRIVENTION, a whole-food, high-fiber, plant-based diet plus behavioral coaching in MGUS/SMM with elevated BMI improved metabolic and inflammatory risk factors, increased microbiome diversity and SCFA production, and stabilized or improved disease trajectories, with Vk*MYC data showing delayed myeloma progression and reinvigorated anti-tumor immunity [50]. A Mediterranean diet intervention in MPNs likewise suggested microbiome and inferred functional shifts consistent with reduced inflammation [70]. These data support further trials testing structured dietary programs as low-risk, microbiome-responsive adjuncts in precursor gammopathies, clonal hematopoiesis, and chronic myeloid neoplasms. More intensive manipulations—such as fecal microbiota transplantation (FMT) and standardized microbial consortia—have shown activity in steroid-refractory intestinal GVHD and decolonization of multidrug-resistant organisms after allo-HSCT, but FMT carries non-trivial risks, including transmission of pathogens and resistance genes [74], and should remain confined to rigorously controlled clinical trials. Routine administration of over-the-counter probiotics or non-targeted prebiotics to neutropenic or transplanted patients cannot be recommended, given limited efficacy and documented probiotic-associated sepsis [75,76]. Future interventional programs will need to carefully balance potential microbiome benefits against infectious, metabolic, and pharmacologic risks, with special caution in profoundly immunocompromised hosts.

In everyday practice, the most broadly applicable microbiota-conscious measures are conservative and supportive: judicious antimicrobial stewardship to avoid unnecessary broad-spectrum or prolonged antibiotic exposure; rational use of other microbiota-toxic drugs, notably long-term proton pump inhibitors; and, where clinically feasible, support for diet quality along Mediterranean or high-fiber lines rather than extreme restrictive patterns. Importantly, such measures can be implemented immediately within standard-of-care pathways, independent of formal microbiome testing, and are aligned with broader principles of good hematologic and transplant practice.

## 5. Conclusions

Current evidence supports a conceptual framework in which hematologic malignancies evolve within a complex host ecosystem that includes the gut microbiota as a relevant, and potentially modifiable, component. The levels of evidence supporting microbiome–disease associations—ranging from preclinical models to human observational and interventional studies—are summarized in Table 6, while disease-specific patterns and proposed mechanisms are illustrated in Figure 2.

Across leukemias, lymphomas, plasma cell disorders, and clonal myeloid neoplasms, a broad profile has been described, characterized by reduced microbial diversity, depletion of short-chain fatty acid-producing commensals, and enrichment of pro-inflammatory or treatment-adapted taxa [2,65,67]. These alterations have been associated with adverse clinical features, disease progression, and outcomes across therapeutic settings, including chemotherapy, immunotherapy, CAR T-cell therapy, and allo-HSCT [48,63]. Mechanistic insights from preclinical and ex vivo studies suggest that microbiota-derived signals and metabolites may influence hematopoiesis, and inflammatory pathways [70]; however, their translation to human disease remains incompletely defined.

The current human evidence base is limited by small, single-center, and predominantly cross-sectional studies; many rely on 16S rRNA surveys with substantial methodological heterogeneity and susceptibility to confounding factors such as diet, geography, prior therapies, antibiotic exposure, and proton pump inhibitors [77,78,79]. Longitudinal sampling around key clinical inflection points remains uncommon [80] and heterogeneity in sample processing and bioinformatics complicates comparisons. These constraints preclude causal inference and currently do not support the routine use of microbiome profiling or empiric microbiota-targeted interventions in clinical practice.

A limitation of this review is the imbalance in the depth of coverage across disease entities, reflecting the uneven availability of data. While acute leukemias and MM are more extensively characterized, evidence for myelodysplastic syndromes, myeloproliferative neoplasms, and clonal hematopoiesis remains comparatively limited.

Meaningful translation will depend on large, prospectively collected, longitudinal, multi-omic datasets embedded within therapeutic and transplant trials and supported by standardized sampling and analytic workflows. Within this framework, pharmacomicrobiomics, microbiome-informed risk stratification, and rational microbiota-targeted interventions represent the most promising avenues for clinical application [9,47].

At present, the microbiota should be considered an integral component of the host context in which hematologic malignancies arise and are treated, rather than an immediately actionable therapeutic target. The integration of microbiome science into clinical research frameworks may ultimately enable more precise risk stratification and biologically informed therapeutic strategies, but translation into routine practice will require robust and reproducible evidence.

## Figures and Tables

**Table 1:** Disease-specific gut microbiota alterations in acute leukemias.

Disease/Setting	Microbiota Features	Key Mechanistic Insights	Clinical Correlations/Outcomes	References
Childhood precursor B-ALL	Reduced α-diversity; depletion of SCFA-producing taxa; enrichment of opportunistic organisms	Altered early-life microbial exposures (“delayed infection” model) → disrupted TH17/TREG balance, impaired immune priming, increased inflammatory tone; microbiota integrates environmental signals affecting pre-leukemic clone progression	May influence progression from pre-leukemic clones to overt ALL; potential target for microbiota-based prevention strategies	[37,38,39]
Adult AML (newly diagnosed, pre-treatment)	Preserved α-diversity; β-diversity shifts: ↑ *Enterococcus*/*E. faecium*, ↑ *Lachnoclostridium*; ↓ *Faecalibacterium*, *Roseburia*, *Collinsella*, *Ruminococcus*, *Agathobacter*; ↓ SCFA (acetate, butyrate)	Dysbiosis alters SCFA-mediated anti-inflammatory signaling; microbial composition correlates with systemic inflammation and hematopoietic niche modulation	High *Enterococcus*/low *Faecalibacterium* associated with unfavorable ELN risk; SCFA and microbial profiling may predict infection risk and chemotherapy toxicity	[23,34,40]
AML/acute leukemias undergoing allo-hsct	Severe α-diversity loss; domination by single taxa (*Enterococcus*, *Streptococcus*, *Proteobacteria*); context-dependent geography: some cohorts show *Bacteroides*, *Akkermansia*, *Escherichia* predominance	Conditioning, antibiotics, mucosal injury → dysbiosis; microbiota diversity loss → pro-inflammatory milieu (IL-6, TNF-α), altered engraftment	Predicts acute/chronic GVHD, infection risk, relapse, non-relapse mortality, overall survival; diversity loss and domination are robust prognostic markers	[42,43,44]

Abbreviations: B-ALL: B-Acute Lymphoblastic Leukemia; SCFA: Short Chain Fatty Acids; AML: Acute Myeloid Leukemia; ELN: European Leukemia Network; GVHD: Graft-Versus-Host Disease.

**Table 2:** Gut microbiota alterations in chronic lymphocytic leukemia (CLL).

Setting/Model	Microbiota Alterations	Mechanistic Insights	Clinical Correlations	References
Treatment-naïve CLL (human, fecal profiling, n ≈ 59–60)	↓ α-diversity; ↓ SCFA-producing taxa (*Lachnospiraceae*, *Ruminococcaceae*, *Bifidobacterium*, *Anaerostipes*); ↑ dysbiosis-associated taxa (*Bacteroides*, *Parabacteroides*, *Sutterella*); ↑ *Firmicutes*/*Bacteroidota* ratio; ↑ *Proteobacteria*	Dysbiosis may impair immunoregulatory TREG/TH17 balance, reduce SCFA-mediated anti-inflammatory signaling, and exacerbate systemic inflammation	Low-diversity cluster associated with more advanced disease, higher prior treatment rates, increased progression events, shorter time to first treatment, unmutated IGHV, high CD38 expression	[45,47]
Experimental CLL model (Eµ TCL1 mice)	Low microbial diversity; dominance of *Mucispirillum* and *Parabacteroides*	Microbiome causally influences leukemia kinetics; low diversity accelerates disease progression	High-diversity microbiota slows leukemia development; supports mechanistic link between gut microbes and CLL pathobiology	[45]

Abbreviations: SCFA: Short Chain Fatty Acids; IGHV: Immunoglobulin Heavy Chain Variable region; CLL: Chronic Lymphocytic Leukemia.

**Table 3:** Gut microbiota alterations in multiple myeloma and precursor states (MGUS/SMM).

Disease/Setting	Microbiota Alterations	Mechanistic Insights	Clinical/Translational Correlations	References
MM (diagnosed)	↓ α-diversity; ↓ SCFA producers (*Ruminococcaceae*, *Lachnospiraceae*, *Faecalibacterium*); ↑ *Proteobacteria*, *Streptococcus*, *Klebsiella*, *Pseudomonas*	Dysbiosis promotes TH17/IL-17-skewed inflammation, IL-6 production, and pro-myeloma microenvironment	Microbial composition correlates with disease stage (ISS), inflammatory milieu, and progression risk	[2]
MGUS/SMM (precursor states)	Early loss of SCFA-producing taxa; expansion of pro-inflammatory taxa (similar patterns as MM)	Microbiota-driven TH17 activation accelerates progression to MM (preclinical vk*myc model)	Higher BM IL-17 levels predict faster progression; microbiome patterns may inform early interventions	[2,48]
Protective/anti-myeloma taxa/metabolites	Urolithin-producing bacteria; butyrate-producing commensals (enriched by high-fiber diet)	Urolithin a cytotoxic to mm cells, inhibits proliferation, synergizes with bortezomib; SCFAs modulate TH17/immune responses	High-fiber diet improves microbiome diversity, SCFA production, reduces inflammatory markers, delays progression (NUTRIVENTION trial, vk*myc model)	[49,50]
Transplant setting (auto-/allo-HSCT in mm)	↓ α-diversity during conditioning/engraftment; transient ↑ *Bacteroides*, *Enterococcus*	Dysbiosis contributes to mucosal toxicity, neutropenic fever, bacteremia	Baseline and on-treatment microbiome features correlate with transplant tolerance, infectious risk, and engraftment kinetics	[2]
Mendelian randomization/genetic causality	Genetically predicted ↑ *Eubacterium Ruminantium* → ↑ MM risk; ↑ *Dorea*, *Coprococcus*, *Ruminococcaceae* Ucg014, *Eubacterium rectale* → protective	Host–microbe configurations may modulate baseline susceptibility	Supports a causal role of specific microbial taxa in mm risk and progression	[51,52]

Abbreviations: MGUS: Monoclonal Gammopathy Of Undeterminate Significance; SMM: Smoldering Multiple Myeloma; MM: Multiple Myeloma; HSCT: Hematopoietic Stem Cell Transplantation; ISS: International Staging System.

**Table 4:** Gut microbiota alterations in lymphomas.

Lymphoma Type/Setting	Microbiota Alterations	Mechanistic Insights	Clinical Correlations	References
MALT lymphoma (gastric)	Infection with *Helicobacter pylori*	Chronic antigenic stimulation drives lymphomagenesis	Eradication therapy induces durable remission in localized disease	[53,54]
EBV-associated lymphomas (Hodgkin, Burkitt, EBV+ DLBCL)	Latent EBV infection in B cells	Viral proteins modulate B-cell survival, immune evasion, cytokine production	EBV positivity linked to specific lymphoma subtypes and progression	[53,54]
DLBCL (primary, treatment-naïve)	↓ α-diversity; ↑ *Proteobacteria* and Enterobacteriaceae; ↓ SCFA-producing taxa (*Faecalibacterium*, *Roseburia*, *Lachnospiraceae*)	Dysbiosis may impair barrier function, modulate immune tone, alter bile acid and xenobiotic metabolism	Reduced diversity correlates with aggressive histology; baseline microbiome predicts R-CHOP response and infectious risk	[55,56,57]
DLBCL (relapsed/refractory)	↑ *Enterococcus*; ↓ *Fusobacterium*; predicted ↑ cytochrome p450 pathways	Microbiota may influence drug metabolism and therapeutic resistance	Shifts during therapy associated with treatment outcomes and toxicity; baseline *Bacteroides fragilis* abundance predicts CAR T response	[57,63]
Follicular lymphoma (FL), treatment-naïve	↑ α-diversity; ↑ *Ruminococcaceae* (*Oscillospirales*); ↓ *Coriobacteriaceae*; functional upregulation of bacterial secretion systems, downregulation of carbohydrate/thiamine metabolism	SCFA production and immune modulation may support tumor growth; high *Ruminococcus* correlates with higher tumor burden	“high-*Ruminococcus*” patients show elevated LDH, trend to higher histologic grade and FLIPI; may reflect immune/metabolic milieu supportive of lymphoma	[57,58]
B-cell NHL (DLBCL, FL, MZL)	General dysbiosis; more pronounced diversity loss in aggressive histologies	Dysbiosis may influence systemic inflammation and immune regulation	Diversity loss correlates with aggressive histology and adverse outcomes	[59]

Abbreviations: MALT: Mucosa-associated Lymphoma Tissue; EBV: Epstein-Barr Virus; DLBCL: Diffuse Large B-Cell Lymphoma; NHL: non-Hodgkin Lymphoma; FL: Follicular Lymphoma; MZL: Marginal Zone Lymphoma.

**Table 5:** Gut microbiota alterations in clonal hematopoiesis of indeterminate potential (CHIP), myelodysplastic syndrome (MDS), and myeloproliferative disorders (MPDs).

Disease/Setting	Microbiota Alterations	Mechanistic Insights	Clinical Correlations	Key References
CHIP (DNMT3A/TET2 mutant)	Age-related dysbiosis; Gram-negative overgrowth; reduced SCFA producers	Gut leakiness and microbial metabolites (ADP heptose) selectively expand mutant HSPCS via ALPK1–NFΚB; effect mitigated by antibiotics targeting Gram-negative bacteria	Pre-leukemic state; increased cardiovascular risk; inflammatory milieu	[68,69]
MDS (high-risk/excess blasts)	Enriched: Gram-negative *Prevotella* spp.; depleted: *Akkermansia*, *Ruminococcus*; shift to LPS-rich communities	Pro-inflammatory signaling; ADP heptose → ALPK1–NFΚB → expansion of DNMT3A/TET2 mutant HSPCS; loss of SCFA-mediated anti-inflammatory effects	Correlates with higher IPSS-R risk category, systemic inflammation, pre-leukemic clone expansion	[65,66,69]
Polycythemia vera (PV)	Reduced α-diversity; depletion: firmicutes (butyrate producers: *Faecalibacterium*, *Ruminococcaceae*, *Lachnospiraceae*)	Reduced SCFA production → enhanced inflammation; microbiota partially normalized under interferon α2 therapy	Correlates with systemic inflammation, thrombotic risk; treatment-specific microbial shifts	[67,70]
Essential thrombocythemia (et, JAK2 V617F-positive)	Pronounced depletion of SCFA-producing *Firmicutes*; increased abundance of taxa linked to inflammation	Loss of anti-inflammatory taxa contributes to chronic inflammation; inferred pro-inflammatory metabolite shifts	Correlates with elevated TNF-α, pro-thrombotic phenotype	[67]
Mixed MPNs (PV, ET, MF)	Altered community structure; reduced anti-inflammatory *Phascolarctobacterium*; variable taxa abundance depending on diet/interventions	Microbiota-inflammation axis; diet-mediated modulation of inflammatory tone	Mediterranean diet reduces inflammation-associated taxa; potential impact on disease trajectory	[52,70]

Abbreviations: CHIP: Clonal Hematopoiesis of Indeterminate Potential; DNMT3A: DNA methyltransferase 3 alpha; TET2: Tet methylcytosine dioxygenase 2; ADP: Adenosine diphosphate; HSCPS: Hematopoietic Stem and Progenitor Cells; ALPK-1: alpha-protein kinase 1; NF-KB: Nuclear Factor kappa-light-chain-enhancer of activated B cells; MDS: Myelodysplastic Syndromes; SCFA: Short Chain Fatty Acids; JAK2: Janus Kinase 2; ET: Essential Thrombocytemia; MF: Myelofibrosis; LPS: lypopolysaccharides.

**Table 6:** Levels of evidence linking gut microbiota to hematologic malignancies.

Evidence Type	Characteristics	Strengths	Limitations	Examples in Hematologic Malignancies
**Animal models**	Controlled experimental systems (e.g., germ-free mice, microbiota manipulation)	Allow mechanistic testing and causal inference	Limited direct translatability to human disease	Pax5^+/−^ ALL model; Vk*MYC myeloma model; microbiota manipulation experiments
**Cross-sectional human studies**	Single time-point microbiome profiling in patients and controls	Provide initial identification of dysbiosis patterns	Cannot determine causality; prone to confounding (diet, antibiotics, treatment)	Studies in AML, CLL, MM describing reduced diversity and altered taxa
**Longitudinal/prospective cohorts**	Repeated sampling over time or before/after treatment	Allow temporal associations and outcome correlations	Still observational; sample sizes often limited	HSCT microbiome studies predicting GVHD and mortality
**Intervention studies**	Dietary or microbiota-targeted interventions	Directly test modifiability of microbiome	Currently few and small; clinical endpoints uncertain	Diet-based interventions (e.g., high-fiber diet in MGUS/SMM)
**Genetic inference approaches**	Methods such as Mendelian randomization	Suggest potential causal links using genetic instruments	Indirect evidence; dependent on GWAS quality and assumptions	MR studies linking microbial taxa to leukemia or MM risk

Abbreviations: ALL: Acute Lymphoblastyc Leukemia; Vk*MYC: variante K MYC; AML: Acute Myeloid Leukemia; CLL: Chronic Lymphocytic Leukemia; MM: Multiple Myeloma; HSCT: Hematopoietic Stem Cell Transplantation; GVHD: Graft-versus-host disease; GWAS: Genome-wide association study; MGUS: Monoclonal gammopathy of undeterminate significance; SMM: Smoldering Multyple Myeloma; MM: Multiple Myeloma; MR: Mendelian randomization.

## Data Availability

No new data were created or analyzed in this study.

## Abbreviations

SCFA, short-chain fatty acid; Chemo, chemotherapy; CAR-T, chimeric antigen receptor T-cell therapy; α-diversity, microbial diversity metric; Th17, T helper 17 cells; IL, interleukin.
